# The Ghrelin-AgRP Neuron Nexus in Anorexia Nervosa: Implications for Metabolic and Behavioral Adaptations

**DOI:** 10.3389/fnut.2019.00190

**Published:** 2020-01-09

**Authors:** Mathieu Méquinion, Claire J. Foldi, Zane B. Andrews

**Affiliations:** Monash Biomedicine Discovery Institute and Department of Physiology, Monash University, Clayton, VIC, Australia

**Keywords:** behavior, anorexia, hunger, appetite, AgRP, GHSR

## Abstract

Anorexia Nervosa (AN) is viewed as primarily a psychiatric disorder owing to the considerable behavioral and genetic overlap with mood disorders and other psychiatric traits. However, the recent reconceptualization of AN as one of both psychiatric and metabolic etiology suggests that metabolic circuits conveying hunger, or sensitive to signals of hunger, may be a critical nexus linking metabolic dysfunction to mood disturbances. Within the brain, hunger is primarily percieved by Agouti-related (AgRP) neurons and hunger increases plasma concentrations of the hormone ghrelin, which targets ghrelin receptors on AgRP neurons to facilitate metabolic adaptations to low energy availability. However, beyond the fundamental role in maintaining hunger signaling, AgRP neurons regulate a diverse range of behaviors such as motivation, locomotor activity, negative reinforcement, anxiety, and obsession and a key factor involved in the manifestation of these behavioral changes in response to activation is the presence or absence of food availability. These changes can be considered adaptive in that they promote affective food-seeking strategies in environments with limited food availability. However, it also suggests that these neurons, so well-studied for their metabolic control, shape mood-related behaviors in a context-dependent manner and dysfunctional control leads not only to metabolic problems but also potentially mood-related problems. The purpose of this review is to underline the potential role of AgRP neurons and ghrelin signaling in both the metabolic and behavioral changes observed in anorexia nervosa. We aim to highlight the most recent studies on AgRP neurons and ghrelin signaling and integrate their metabolic and behavioral roles in normal function and highlight how dysfunction may contribute to the development of AN.

## Neuroendocrine Control of Energy Homeostasis

Energy homeostasis is the balance between energy intake, including the total amount and density, and energy expenditure, including basal metabolic rate, diet-induced, and activity-induced thermogenesis. The maintenance of energy homeostasis is an integral process required for the ongoing sustainability and survival of a species, especially since starvation leads to death and metabolic imbalance affects reproductive fertility. The hypothalamus is a key structure involved in the maintenance of energy homeostasis and is composed of different nuclei that contain a variety of neuronal populations. These nuclei are involved in vital functions such as stress, thermogenesis, reproduction, growth, metabolism, and food intake (1). Key nuclei responsible for energy homeostasis include the arcuate nucleus (ARC), the ventromedial hypothalamic nucleus, the paraventricular hypothalamic nucleus, the lateral hypothalamus and the dorsomedial hypothalamic nucleus (2, 3).

The ARC was first implicated in the control of food intake and glycemia using neurotoxic drug injections (4, 5). Indeed, it was later discovered that these treatments led to the destruction of specific neuronal populations including proopiomelanocortin (POMC)-expressing neurons and agouti-related peptide (AgRP)-expressing neurons. AgRP neurons, the focus of this review, are critical for survival since adult-ablation of these neurons leads to anorexia, rapid weight loss, and death by starvation (6). Further, beyond the fundamental role in maintaining hunger signaling, AgRP neurons regulate a diverse range of behaviors such as motivation, locomotor activity, negative reinforcement, anxiety, and obsession (7–10), and a key factor involved in the manifestation of these behavioral changes in response to activation is the presence or absence of food availability. For example, AgRP activation in presence of food drives food intake, whereas when food is not available it drives other motivated goal-directed behaviors and reduces anxiety-like behaviors (7, 8, 11, 12). These changes can be considered adaptive in that they promote affective food-seeking strategies in environments with limited food availability. However, it also suggests that these neurons, so well-studied for their metabolic control, shape mood-related behaviors in a context-dependent manner and dysfunctional control leads not only to metabolic problems but also potentially mood-related problems.

AgRP neurons are located at the base of the third ventricle near the median eminence and can rapidly sense changes in metabolic state through neuroendocrine feedback mechanisms involving various hormones and nutrients. During hunger or energy deficiency, where the body expends more energy than it receives, elevated plasma ghrelin provides critical feedback information to the brain, signaling negative energy balance (13). Indeed, AgRP neurons are a key target of plasma ghrelin, with >80% of AgRP (coexpressing Neuropeptide Y [NPY]) neurons also expressing the ghrelin receptor (GHSR; growth hormone secretagogue receptor) (14). Moreover, a number of functions ascribed to ghrelin can be attenuated or blocked when manipulating GHSRs in the ARC or after deleting GHSRs from AgRP neurons (15, 16). Thus, in a physiological setting many of the behavioral adaptations caused by AgRP activation maybe be related to ghrelin signaling.

A primary and critical role of ghrelin is to inform the brain of low energy availability. Although GHSRs are found in a number of different brain regions, AgRP neurons remain a primary target to convey this metabolic information via a variety of specific projections (17). Ghrelin-AgRP feedback is specifically designed to prevent excessive and pathological weight loss. This system, however, is not fail-safe, with AN a prominent example whereby patients present with a severe energy deficit and dangerously low body weight.

AN belongs to a family of eating disorders including bulimia nervosa and binge-eating disorder. The pathogenesis of AN involves a number of genetic, neurobiological, psychological, socio-cultural, and developmental factors (18) with accumulating evidence suggesting an important role for metabolic dysfunction (19, 20). Further support for the metabolic origins of AN comes from a recent genome-wide association study that revealed significant genetic correlations with metabolic traits including insulin resistance and glucose metabolism (21). AN patients present various hormonal and neurobiological alterations associated with negative energy balance, leading to the dysregulation of homeostatic systems (22, 23), which is frequently associated with other psychiatric disorders (24–26). Given that AgRP-ghrelin signaling influences both metabolic and behavioral consequences, particularly in the absence of food availability, it is intriguing to speculate that abnormal function of this system may contribute to both the metabolic and behavioral consequences of AN. The purpose of this review is to underline the potential role of AgRP neurons and ghrelin signaling in both the metabolic and behavioral changes observed in AN. We aim to highlight the most recent studies on AgRP neurons and ghrelin signaling and discuss their metabolic and behavioral roles in normal function and discuss how dysfunction may contribute to the development of AN.

## AN: Prevalence and Persistence

The first description of behaviors linked to AN date back the Middle Ages with the case of St Catherine of Siena (27), although it was Sir William Gull who first coined the term AN in 1874 to define a number of his patients (28). Diagnostic criteria for AN comprise persistent restriction of food intake leading to significantly low body weight in the context of what is minimally expected for the height, age and developmental stage of the individual, in addition to a fear of weight gain and becoming fat, and a disturbance of the self-body perception with dysmorphobia. Different studies report the incidence of eating disorders including AN among the Australian or European populations, as <1–5% of the population, and predominantly in females (29–31). AN has long-term and long-lasting effects, as evidenced by a large cohort study following inpatients over 25 years that showed remission in only 30% of patients, with close to 46% in either partial remission or with a crossover diagnosis of eating disorder not otherwise specified (EDNOS) and 16% of patients retaining their AN diagnosis (32).

## Endocrine Consequences of AN

Many of the endocrine alterations observed in AN patients are found in all animals in response to prolonged fasting or food restriction in order to meet and maintain energy demands (33, 34). Different phases are classically described in mammals, including humans (33, 35); following a hypoglycaemic period the secretion of glucagon, epinephrine or glucocorticoids, the main counter-regulatory hormones, lead to a glucose overcompensation from glycogenolysis and then gluconeogenesis mainly from the liver and kidney. Fasting is accompanied by other hormonal alterations including a decrease of plasma leptin and insulin concentrations, in parallel with the increase of plasma ghrelin concentrations. If the fasting is prolonged the organism starts to use stored lipids, causing a marked increase of glycerol and free fatty acids in the plasma, both of which are used by the liver to produce glucose and ketone bodies, respectively (35). Lipolysis, gluconeogenesis and synthesis of ketone bodies caused by severe restriction are all associated with a reduction of energy expenditure, as a means to protect energy stores (36–38). Finally, when lipid stores are completely depleted, an organism enters a proteolytic phase in which proteins from the muscle provide carbon precursors used in the different steps of gluconeogenesis (35). This depletion of energy stores is associated with a reduction of both lean and fat mass and in the most severe situations induces muscle wasting as well as decreased body temperature (39, 40). AN patients exhibit most, if not all, of these physiological consequences of severe calorie restriction, however, it is interesting to note that the BMI used to reflect the severity of the pathology is regularly lower in AN patients compared to starvation and/or food restriction studies in healthy volunteers. For example, it is not rare to observe a BMI lower than 15 kg.m^−2^ in AN patients at admission whereas subjects from the seminal Minnesota semi-starvation study presented as 16.4 kg.m^−2^, on average, after 24 weeks of food restriction (20, 41, 42). This is likely due to the paradoxical increase in energy expenditure that manifests in the majority of AN patients (43, 44). Interestingly, no relationship has been found between the severity of the disease and mood disorder outcomes, although lower bone mass density was observed in more severe cases (41, 42, 45).

Among all the hormones affected in AN patients, changes in leptin and ghrelin may be best used to aid in diagnosis (18, 24, 46, 47). Some euglycemic hyperinsulinemic clamp studies in AN patients have shown significantly lower total ghrelin, suggesting an increase of satiety sensation (48). Other studies have suggested that despite the high levels of plasma ghrelin in AN patients, ghrelin resistance could explain the ability to engage in persistant food restriction (49–52). Significant elevations in plasma AgRP levels have been demonstrated in AN patients compared to controls (53) and subtle impairments in cognitive flexibility associated with acute AN were negatively correlated with plasma AgRP levels (53). Moreover, several genetic, and genome-wide association studies have shown associations between the occurrence of AN and ghrelin-related hormones and peptides including preproghrelin, ghrelin O-acyltransferase (GOAT), the enzyme required for acylation, and AgRP (54–57). Genetic evidence from patients supports a role of AgRP in AN, indicating that allelic variations in the AgRP gene are associated with susceptibility to AN, with one polymorphism conveying a relative risk of 2.63 for carriers to develop the condition (58). Single nucleotide polymorphisms in the melanocortin-3 receptor (MC3R) were proposed to underlie this association, however, direct sequencing of four single nucleotide polymorphisms in the MC3R did not demonstrate significant associations with AN (59).

## Behavioral Changes in AN

AN is often associated with comorbid diagnoses, particularly anxiety and depression (60, 61). Other psychiatric tendencies such as obsessive-compulsive behavior and harm avoidance have also been observed in many patients (60, 61). Besides restrictive feeding behavior, up to 80% of AN patients engage in excessive physical activity in order to reduce their body weight, a behavior that is often considered compulsive (62). In contrast, non-AN subjects that participated in the Minnesota semi-starvation study reported lethargy and a reduction of self-initiated spontaneous activity.

Although the mechanisms need to be clarified, these results suggest homeostatic hunger signals, such as AgRP neuronal activity and plasma ghrelin, may manifest different goal-directed behavioral outcomes in AN patients compared to healthy controls. Both AgRP neuron activity and ghrelin signaling increase motivation, which is usually directed toward a food goal. However, when food is no longer a relevant goal, a shift in goal-directed behavior to locomotor activity may reflect a strategy to channel motivation derived from homeostatic signaling toward non-food related outcomes. In support of this, both AgRP neuron activity and ghrelin signaling increase locomotor activity in rodents when food is unavailable (63–66) and blocking ghrelin/AgRP actions decreases physical activity and/or food anticipatory behavior compared to control animals (67, 68). Moreover, in time-schedule feeding studies, ghrelin is required to promote food anticipatory activity (69, 70) and plasma ghrelin concentrations are positively correlated with food anticipatory activity. Central ghrelin injection also increased anticipation of palatable food (71).

It is regularly reported that stressful life events (e.g., separations, violence, aggression) precede the development of eating disorders (52). Many studies show that perinatal or juvenile stress can predispose individuals to the development of metabolic phenotypes in humans and in rodents (72, 73) and contribute to psychiatric phenotypes (74, 75). These studies highlight that perinatal and/or juvenile stressors can manifest in adulthood as both metabolic and psychiatric problems, reinforcing the important link between metabolic and mood related circuits in the brain. Thus, we put forward the novel hypothesis that early-life stress might impact common neural circuits regulating energy homeostasis and emotional mood responses, which could predispose individuals to both metabolic and psychiatric problems in later life.

## The Ghrelin-AgRP Neuron Axis in Animal Models of AN

The homozygous anx/anx mouse model develops the primary symptom of AN, starvation and subsequent emaciation, however dies prematurely around 3 weeks of age, when they weigh around half as much as their wildtype siblings and display a range of hypothalamic neuropeptidergic and molecular aberrances (76), including an increased number of AgRP/NPY immunopositive cell bodies in ARC (77). However, the neuronal circuits responsible for energy homeostasis are not fully developed during the short lifespan of this model, making it difficult to extrapolate these findings to the neuroendocrine dysfunction observed in AN patients. Although ghrelin resistance is known to occur in obese animal models (78–81), to our knowledge, no study has directly implicated altered plasma ghrelin levels in the anx/anx phenotype or in other animal models of AN. In the activity-based anorexia (ABA) rat model, which relies on allowing animals unhindered access to running wheels in combination with time-limited access to food (82), central infusion of the inverse agonist AgRP (83–132) increased both cumulative food intake and basal body temperature during exposure to ABA conditions, but did not significantly impact body weight loss (83).

In support of the hypothesis that early-life stress might contribute to the development of AN, it has been shown that early-life stress in a mouse model impacts on both leptin and ghrelin secretion and AgRP fiber density, with changes in plasma ghrelin seen only in females (84). Importantly, both ghrelin and leptin play a critical role in the development of hypothalamic circuits regulating feeding and diet-induced obesity impairs hypothalamic NPY and AgRP signaling, as well as POMC fiber pathways (84, 85). Thus, early-life stress can impact on neural circuits controlling energy homeostasis and can predispose individuals to metabolic disease (diet-induced obesity) in adulthood (86–88). Whether or not similar early-life stress events predispose to AN in animal models via homeostatic circuit modification has not been addressed but should be considered in the future.

## The role of Ghrelin and AgRP Neurons in Metabolism

AgRP neurons are essential hunger-sensing neurons, as shown by the seminal studies of Luquet et al. (6). In this study, the authors used mice expressing the human diphtheria toxin receptor in AgRP neurons (AgRP^DTR^ mice) allowing the destruction of these neurons after diphtheria toxin treatment. Diphtheria toxin in adult mice caused a rapid and substantial decrease in food intake and body weight, results that have been subsequently confirmed using similar techniques (89, 90). Importantly, neonatal ablation of AgRP neurons did not lead to a pronounced phenotype (6). These results highlight not only the importance of compensatory mechanisms in the neurodevelopmental process of hypothalamic feeding circuits but also the indispensable role of the AgRP neurons in sensing hunger and feeding behavior. As a key hunger signal, ghrelin targets AgRP neurons to increase food intake and although it has been shown that ghrelin requires AgRP neurons to increase food intake, a number of studies demonstrate that other ghrelin sensitive regions, including the hippocampus and brainstem, are also involved in the control of food intake (91–95).

Chemogenetic and optogenetic techniques developed more recently have allowed researchers to comprehensively define this role of hunger-sensing AgRP neurons (8, 9, 96–99). By using DREADD hM3Dq expression in AgRP neurons of NPY and GABA receptor double knockout mice, Krashes et al. (98) showed that both NPY and GABA are necessary for a rapid increase of food intake, whereas stimulating AgRP neurons in the absence of NPY and GABA had a delayed effect on food intake indicating AgRP peptide produces a slower feeding effect than NPY or GABA (98). Besides stimulating food intake, activation of AgRP neurons increases fat mass and reduces energy expenditure, respiratory exchange ratio and body temperature, all of which contribute to the conservation of energy (63, 100, 101).

Rodents, like humans, adopt similar strategies to cope with acute or chronic energy deficit in order to maintain vital signs in homeostatic range and organ functions (102, 103). At the level of AgRP neurons, food deprivation leads to changes in gene expression in pathways involved in hormone signaling, including leptin, insulin and ghrelin that leads to modulation of AgRP, NPY and GABA expression (104). Ghrelin acts on central and peripheral targets via the expression of GHSR1a and, as well as increasing food intake, ghrelin reduces energy expenditure and fat usage, increases glycogenolysis and glycemia (47). Collectively, ghrelin is a metabolic signal that informs the brain of low energy availability, allowing for metabolic adaptations to conserve energy. Ghrelin action via the GHSR1a on AgRP neurons is partially responsible for its effect on food intake, but expression of GHSR also acts to normalize glycemia under fasted and food restricted conditions via effects on plasma glucagon and an upregulation of gluconeogenesis gene expression (105). Along with other similar findings on feeding and glycemia (16), these results suggest that ghrelin acts via the GHSR in AgRP neurons primarily to control glycemia in response to negative balance, with a secondary effect on feeding. Consistent with these physiological studies, the GHSR is expressed by a large majority of AgRP neurons (90%) and a significant portion of Growth hormone releasing hormone neurons (25%) and chemogenetic inhibition of GHSR neurons in the mediobasal hypothalamus blocks fasting-induced feeding, whereas chemogenetic activation increases food intake in satiated mice (15). Also highlighting the importance of the ghrelin-AgRP nexus is the ability of plasma ghrelin to rapidly enter the ARC for sensing by ARC (AgRP) neurons. In fact, this is the most prominent site for plasma ghrelin entry into the brain and accessibility increases during energy deficit (106–108). Taken together, these findings underline the important interaction between ghrelin and AgRP neurons that is magnified in situations of energy deficit such as AN. Indeed, AgRP neurons are required to integrate signals of energy status for the normal action of ghrelin, as we recently showed that glucose-sensing via AMPK in AgRP neurons modulates the ability of ghrelin to stimulate food intake (109).

AgRP neurons are important to sense and compute incoming information related to energy availability, a process that involves both sensory detection from olfactory and visual cues (12), as well as metabolic feedback in response to food consumption (110–112). Fiber photometry to visualize AgRP population activity showed a rapid reduction in fasted AgRP activity (within seconds) in response to the presentation of food, with a greater reduction in response to highly palatable foods (12). The reduction in AgRP activity was sustained only if food remained available for consumption after presentation and AgRP activity returned to high fasted levels if food was inaccessible or removed after presentation (12). Su et al. showed that sustained reductions in AgRP neurons required gastro-intestinal nutrient and hormonal feedback over a longer timescale (30 min) (110, 111). These results demonstrate that AgRP neurons are responsive to different feedback modalities over different time frames—sensory feedback occurs within seconds and predicts the value of incoming nutrients, whereas nutrient and hormonal feedback occurs over minutes and provides a post-ingestive confirmation of actual calorie consumption to sustain changes in AgRP feedback. We recently showed that carnitine acetyltransferase (Crat) in AgRP neurons is an important enzyme required for the normal response to calorie intake during fasting, calorie restriction and restricted feeding (112–114), highlighting that normal metabolic processing of AgRP neurons is required to detect and compute calorie feedback.

Interestingly, signals of long term energy storage, such as leptin from adipose tissues, provides feedback to control AgRP neuronal activity over hours to days (110). Each aspect of the temporal feedback model may be important for normal homeostatic and behavioral actions of AgRP neurons and ghrelin, as a hormone that increases AgRP activity. If adipose stores are depleted, the absence of long-term feedback from leptin may affect both the sensory (seconds) and homeostatic (minutes) response to food. Indeed, AN is characterized by a loss of both long-term and homeostatic post-ingestive responses due to both the lack of food intake and absence of leptin, which has significant impact on the sensory control of AgRP. As a result, this may impair immediate behavioral and stress responses, something that is often reported in AN patients.

## AgRP and Ghrelin Signaling Impact on Behavior

Optogenetic stimulation of hypothalamic axon terminals in the paraventricular hypothalamic nucleus, lateral hypothalamus, and in extra-hypothalamic axon terminals in bed nucleus of the stria terminalis, paraventricular thalamus, and medial amygdala increase food intake (8, 10, 17, 97, 99, 115). An intriguing observation is that there are a number of brain regions innervated by AgRP neurons that have no effect on food intake or other metabolic parameters (17). In addition, a number of the brain regions innervated by AgRP neurons that increase food intake also play important roles in the modulation of mood and motivation, including the output regions of the hypothalamus described above. Thus, AgRP neurons, as key neurons detecting hunger, are anatomically connected to numerous brain regions to control both feeding-related and non-feeding related behaviors.

Besides food intake, acute activation of AgRP neurons drives motivation to obtain food rewards, food-seeking locomotor behavior and a number of peripheral changes to limit energy expenditure (11, 63). In addition, AgRP neuronal activation is shown to evoke stereotypical behavioral patterns including repetitive obsessive and compulsive tendencies (9) when food was not available for consumption, similar to symptoms of AN. Optogenetic activation of AgRP neurons initiates a conditioned place aversion when food is not available, suggesting that increased motivation after AgRP neuronal activation is driven by the desire to remove the aversive feeling, otherwise known as negative reinforcement (11). Notably, fasting, ghrelin and AgRP activation all increase exploratory and risk-taking behavior in order to access food (7, 10, 116, 117). An important distinction here is that food is available during the task if the mouse is willing to risk obtaining it. Taken together, these data establish that AgRP neurons drive a neural signal of hunger, but if this neural signal of hunger is not fulfilled by appropriate food intake, or accessibility to food, this leads to non-feeding behaviors such as obsessive and compulsive tendencies and hyperlocomotion; that is, increased motivation driven by negative reinforcement. Such a response to hunger in the absence of food intake could underlie behavioral changes seen in AN, such as increased motivation for locomotion (exercise) rather than food (118).

Hunger-sensitive AgRP neurons and ghrelin feedback regulate non-food related behaviors, such as mood and motivation, which may be a result of an interaction between the ghrelin-AgRP nexus and stress pathways. This interaction can be bidirectional whereby fasting may activate the ghrelin-AgRP nexus to influence the Hypothalamo-Pituitary-Adrenal (HPA) stress axis (119, 120) or the HPA stress axis affecting the ghrelin-AgRP nexus (121). This interaction is pertinent, since AN patients show increased activation of the HPA stress axis at both the neuroendocrine (increased corticotropic-releasing hormone) and endocrine level (increased cortisol) (122–124), both of which are broadly implicated in neuropsychiatric disease (125). However, it should be noted that ghrelin can also directly activate corticotropic-releasing hormone neurons independently from the ARC (126, 127), indicating that behavioral changes associated with high ghrelin may simultaneously, yet independently, occur at the ARC and paraventricular hypothalamic nucleus.

Nevertheless, all psychological or physical stressors increase plasma ghrelin (128) and ghrelin regulates the HPA axis at the level of the pituitary and hypothalamus (129). The HPA axis mediates the body's response to stressors and facilitates the appropriate mechanisms to deal with stressful events (128). However, dysregulation of the HPA axis can prove maladaptive by promoting mood disorders, such as anxiety, depression, and compulsion (130), or metabolic disorders such as overeating and excessive weight gain (131, 132). In terms of regulating mood, GHSR signaling reduces anxiety and depression-like symptoms in a model of chronic social defeat (133, 134) and a Leu72Met gene polymorphism in the human *ghrelin* gene associates with major depression (135). In response to acute stress, ghrelin regulates the HPA axis to limit anxiety-like behavior (128, 129). However, this appears to be related to the ratio of acyl ghrelin to des-acyl ghrelin since mice lacking the enzyme that acylates ghrelin (GOAT) show increased anxiety-like behavior under both non-stressed and stressed conditions, which not due to changes in corticosterone (136). In addition, there is an unusual paradox, as a number of publications have reported that ghrelin promotes anxiety (137–139). In these studies, animals underwent behavioral testing within 30 min of ghrelin injection without food availability, suggesting that the unfulfilled hunger signal from ghrelin may have promoted an anxiety-like state during the testing period.

GHSR signaling in the brain also influences motivation for food rewards in models of conditioned place preference and operant conditioning (91, 140–143). It is particularly relevant that GHSR signaling in the brain may link stress/mood with the motivation to obtain food reward. For example, chronic social defeat stress in mice drove consumption of high fat diet and weight gain in GHSR wild-type but not GHSR knockout mice (131). Moreover, we have demonstrated that a ghrelin injection conditions a rewarding experience when paired with food availability but conditions an aversive experience when food is withheld (81), similar to examples above showing that AgRP neuronal activation in the absence of food drives a conditioned place aversion. Thus, plasma ghrelin, as a hunger signal from the body, influences mood and motivation and the behavioral readout depends on food availability.

How hunger states can affect mood and motivated behaviors needs addressing when we consider the co-morbidity between metabolic dysfunction and mental illness (144). Moreover, exactly where in the brain both metabolic and mood/motivation circuits interact remains unknown. One important region may be the amygdala given its roles in emotional learning, cue-predicted learning, anxiety, reward processing, and motivation (145). Indirect evidence shows that ghrelin regulates the activity of neurons in the medial amygdala after acute stress (38) and GHSR signaling in the basolateral amygdala regulates neuronal activity in a model of cue-potentiated feeding (146). Furthermore, repeated ghrelin agonist injections in the basolateral amygdala increased fear memory (147). In terms of AN, brain-imaging studies show differential activation of the amygdala in AN patients relative to controls (148) and homeostatic signals such as ghrelin, AgRP and NPY are all significantly increased in AN patients (149, 150). Interestingly, AN patients have significantly higher plasma ghrelin concentrations compared to constitutively lean women (151) and constitutional thinness is not associated with psychological disturbances, amenorrhea, or other hormonal abnormalities associated with undernutrition (36, 152). The mechanisms underlying this difference may be related to increased exercise often observed in AN patients, since exercise is known to increase plasma ghrelin concentrations (153). It is therefore plausible that persistent high levels of plasma ghrelin may contribute to mental health issues in AN patients.

AN patients have other behavioral maladaptations/disturbances not apparently linked to hunger sensing (via AgRP neurons) or hunger signaling (via ghrelin or GHSR). These behaviors include disrupted sleep-wake structure and quality with lower slow wave sleep and rapid eye movement sleep, in addition to harm avoidance and social interaction deficits (154–156). Food restriction protocols in rodents are known to disturb the normal light-dark cycle activity in mice, as shown by food anticipatory activity and a recent study indicating that optogenetic AgRP neuronal activation increased the number and length of wake periods and the duration of non-rapid eye movement (NREM) sleep periods (157). Conversely, chemogenetic inhibition of these same neurons has no effect in satiated mice but reduced NREM sleep and microarousals during NREM sleep in fasted mice (157). Thus, persistently high AgRP and ghrelin levels as seen in AN (149, 150), may also impact behavior via impairing the quality of sleep.

## Conclusion

**Figure 1:**
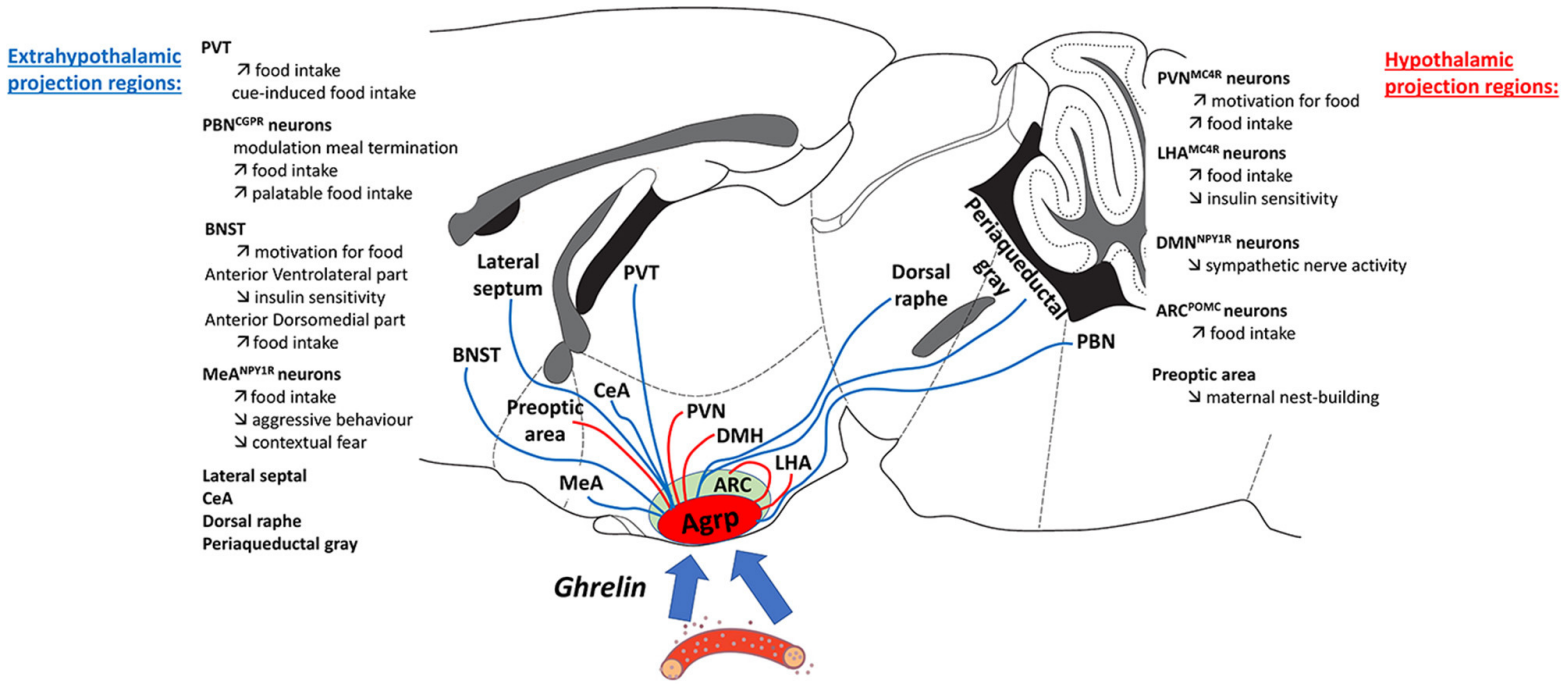
Ghrelin is secreted from the stomach primarily under conditions of negative energy balance and acts to inform the brain of low energy availability. As a signal of energy deficit, ghrelin promotes behaviors to encourage food-seeking and food intake as well as adaptive strategies to cope with hunger, and influence metabolism to maximize energy storage. One of the major targets of circulating ghrelin is the population of AgRP neurons that reside in the arcuate nucleus of the hypothalamus. Ghrelin reaches AgRP neurons and fasting increases permeability to allow greater diffusion of ghrelin into this central target. As highlighted in this figure, AgRP neurons project to a large number of different nuclei throughout the hypothalamus, amygdala, brainstem, thalamus, and midbrain. However, not all AgRP neurons projections stimulate food intake, it is currently thought that only the Agrp to PVN, LHA, BNST, PVT, PBN, and MeA projections influence food intake. Thus, it is important to appreciate that activation of hunger-sensing AgRP neurons affects both feeding and nod feeding pathways when active. Another important observation is that ghrelin and fasting both increase AgRP neuron activity, leading to increased food intake when food is available; whereas when food is unavailable, the activation of AgRP neurons leads to changes in energy metabolism and behavioral adaptations. Such behavioral changes in the absence of food include obsession-compulsion, mood-changes, motivation, aversion, sociability, although the specific circuits involved in these behaviors remain to determined. In situations of acute energy deficit, these behavioral responses are thought to be adaptive, however the consequences of long-term energy deficit on these behavioral response remain unknown. These observations highlight a potential role for disrupted or prolonged chronic ghrelin-AgRP signaling in the absence of appropriate food intake to have a significant impact on normal behavior in anorexia nervosa (AN), a disorder characterized by a severe and chronic energy deficit. Indeed, similar behavioral features have also been observed in patients with AN, therefore, understanding how Ghrelin-AgRP neuronal signaling mediates behavioral and metabolic adaptations in the presence or absence food availability may shed light on the role of these circuits in the pathophysiology of AN.

An understanding of how hunger signals influence mood and motivation may provide valuable insight into the pathogenesis of both metabolic dysfunction and mental illnesses, such as AN. Indeed, AN is viewed as primarily a psychiatric disorder owing to the considerable behavioral and genetic overlap with mood disorders and other psychiatric traits (158). However, the recent reconceptualization of AN as one of both psychiatric and metabolic etiology (19, 20) suggests that metabolic circuits conveying hunger, or sensitive to signals of hunger, may be a critical nexus linking metabolic dysfunction to mood disturbances (see Figure 1). In line with this line of reasoning one would expect that dampening down persistent signals of hunger (AgRP neurons or GHSR activity) may alleviate some potential psychiatric problems associated with AN. However, this would be considered controversial and require substantial experimental evidence to support such actions.

The advent of new technologies developed this last decade has brought with it a new suite of information regarding the activity and function of AgRP neurons within hypothalamic and extrahypothalamic circuits. These neurons appear to be sensitive to a wide range of signals including food cues, nutrients and hormones and respond to these signals (8, 159). In light of this, it is clear that the AgRP neurons may have a significant role in AN at both a metabolic and behavioral level. Future studies are required to examine the causal role of hunger-sensing AgRP neurons and the hunger signal, ghrelin, in behavioral changes associated with AN. A major limitation at this stage, due to the complexity of the etiology of the disease, is an appropriate animal model in which to do so. Novel translational models should incorporate both voluntary reduction in food intake and excessive exercise behavior, both essential elements of body weight loss in AN, in addition to genetic, metabolic and environmental drivers of the human condition.

## Author Contributions

MM wrote the first draft of the manuscript. ZA wrote sections of the manuscript. All authors contributed to manuscript revision, read, and approved the submitted version.

### Conflict of Interest

The authors declare that the research was conducted in the absence of any commercial or financial relationships that could be construed as a potential conflict of interest.
